# GWAS combined with transcriptomics revealed key regulatory genes for inflorescence traits and fruit set rate in Litchi (*Litchi chinensis* Sonn.)

**DOI:** 10.1186/s12864-025-11796-3

**Published:** 2025-07-26

**Authors:** Fachao Shi, Kan Huang, Yonghua Jiang, Hailun Liu, Yingjie Wen, Qian Yan

**Affiliations:** 1 Institute of Fruit Tree Research, Guangdong Academy of Agricultural Sciences; Key Laboratory of South Subtropical Fruit Biology and Genetic Resource Utilization, Ministry of Agriculture and Rural Affairs; Guangdong Provincial Key Laboratory of Science and Technology Research on Fruit Trees, Guangzhou, 510640 China; 2 Key Laboratory of Arable Land Conservation (Middle and Lower Reaches of Yangtze River), Ministry of Agriculture, Micro-elements Research Center, College of Resources & Environment, Huazhong Agricultural University, Wuhan, 430070 China

**Keywords:** Flowering phenotype regulation, Litchi inflorescence development, SNP associations in Litchi, Transcriptomics analysis, Candidate gene identification

## Abstract

As an important tropical and subtropical fruit, litchi’s inflorescence size and the number of florets per inflorescence are crucial factors affecting fruit set rate and yield. This study extensively collected a total of 219 litchi germplasm resources, including those from China and 11 other countries worldwide, systematically evaluating eight core phenotypic traits: inflorescence length (IL), inflorescence width (IW), number of secondary lateral inflorescences (NSLI), number of inflorescence internodes (NII), base to main axis length (BMAL), inverted 5th internode length (I5IL), number of female flowers per inflorescence (NFFI), and fertilization rate (FR). The research findings indicated significant correlations between these agronomic traits and fruit set rate, particularly with high fruit set rate litchi varieties showing notably lower trait expressions in IL, NSLI, NFFI, and NII compared to low fruit set rate varieties. Furthermore, through genome-wide association studies (GWAS), significant SNP loci were successfully identified for the two key traits, NSLI and NFFI, while other traits did not show significant associations. Transcriptome results revealed that differentially expressed genes between two typical inflorescence litchi varieties were mainly enriched in molecular function categories such as catalytic activity and transferase activity, affecting metabolic pathways and secondary metabolite synthesis. Joint analysis of GWAS and transcriptomics suggested that the NSLI-associated gene LITCHI016073 (UBP1-associated proteins) might regulate inflorescence development by influencing gibberellin signaling, while NFFI -associated genes LITCHI019855 (Solute carrier family), LITCHI011125 (SEC3A), LITCHI025977 (Acid phosphatase), and LITCHI023264 (Enolase) affected the development and number of female florets. The functions of these genes were further validated by transcriptome results. qRT-PCR analysis showed that in the Houxian variety (dense inflorescence), the expression levels of LITCHI016073, LITCHI011125, LITCHI025977, and LITCHI023264 during the three critical flowering stages were significantly higher than those in the Edanli variety, while the expression level of the LITCHI019855 gene was significantly lower, strongly demonstrating the important roles of these genes in inflorescence development and fruit set rate regulation. By employing large-scale sample analysis and multi-omics technologies, this study systematically unraveled the intrinsic relationships between litchi inflorescence traits and fruit set rate, providing a solid scientific basis and novel insights for litchi breeding practices.

**Supplementary Information:**

The online version contains supplementary material available at 10.1186/s12864-025-11796-3.

## Introduction

*Litchi chinensis* Sonn., commonly known as lychee or lychee nut, originated in the northern tropical and southern subtropical regions of southern China, with its cultivation history dating back to 211 BC [1]. Currently, the National Litchi Germplasm Resource Bank has recorded over 700 litchi germplasm resources across seven major litchi production areas in China (Guangdong, Guangxi, Fujian, Hainan, Yunnan, Sichuan, and Taiwan) as well as in 11 other countries including India, Bangladesh, Nepal, Vietnam, Thailand, Israel, Australia, Madagascar, South Africa, Mauritius, and Brazil, making it the most complete and distinctive litchi germplasm repository in China with abundant resources. Compared to banana [2], apple [3], and citrus [4], litchi is considered a low-yielding fruit. It is generally believed that litchi production is influenced by multiple factors, including environmental factors such as climate, temperature, and rainfall, as well as other factors like pollination, poor fertilization, and imbalances in the supply and demand of photosynthetic assimilates among different tissues [5–7]. Thus far, numerous studies have assessed external factors related to litchi flowering [8], morphological changes [9], and biochemical mechanisms [10]. Relevant research indicated that phenotypic traits of inflorescences, including the number of female flowers per inflorescence and the female flower fertilization rate, are closely related traits during the flowering process of litchi [11, 12]. In crop production, the optimization of inflorescence structure and floret number is widely recognized as one of the important strategies to enhance yield. For instance, in rice (*Oryza sativa* L.), the number of florets directly affects the number of grains per panicle, which subsequently influences the total yield. Research indicates that increasing the number of grains per panicle and optimizing the panicle structure through breeding techniques can significantly improve rice yield [13]. Similarly, in wheat (*Triticum aestivum* L.), floret degeneration is one of the key factors affecting yield. By regulating nutrient distribution, improving field management, and using plant growth regulators, floret degeneration can be reduced, the number of grains per ear increased, and thereby wheat yield enhanced [14]. In corn (*Zea mays* L.), floret number and inflorescence structure also have a significant impact on yield. The female ear of corn consists of multiple spikelets, each containing several florets. Studies show that increasing floret number, reducing floret degeneration, and optimizing female ear structure are effective ways to improve corn kernel yield and quality [15]. Furthermore, through gene editing techniques such as the CRISPR/Cas9 system, key genes regulating floret development can be modified to directly improve inflorescence structure and floret number, thereby enhancing crop yield [16]. Additionally, the influence of inflorescence structure and floret number on soybean (*Glycine max* L.) yield has also garnered significant attention. The inflorescence of soybean consists of multiple pedicels and flowers, with the number of flowers directly determining the number of pods and ultimate seed yield. Through genetic improvement and field management measures, the number of flowers on soybean plants can be increased, flower abscission reduced, and soybean yield significantly improved [17].

In recent years, with the rapid development of high-throughput sequencing technologies, genome-wide association studies (GWAS) and transcriptome sequencing have become important tools for elucidating complex genetic traits and molecular mechanisms [18–20]. GWAS identifies gene variants associated with specific phenotypes across the entire genome by comparing single nucleotide polymorphism (SNP) variations among different individuals [21, 22]. In recent years, several studies have utilized GWAS to analyze the impact of agronomic traits on tomato quality and disease resistance [23], the influence of sorghum agronomic traits on phenolic content [24], the association between chickpea agronomic traits and drought resistance [25], the relationship between peach fruit traits and aroma content [26], the correlation between jujube traits and its yield and variety characteristics [27], and the association between cherry traits and its yield and quality characteristics [28]. However, the regions identified by GWAS are often large and contain multiple candidate genes, making it difficult to directly determine the functional genes. Transcriptome sequencing, on the other hand, provides rich information at the gene expression level and reveals key genes and their regulatory networks by comparing gene expression profiles across different samples or conditions [29–32]. Therefore, combining GWAS with transcriptome sequencing has become an important strategy for elucidating complex genetic traits. In plant research, the integration of GWAS and transcriptome sequencing has been widely applied to the analysis of important agronomic traits such as yield [18], stress resistance [33], and quality [34]. For example, in rice [35], wheat [13, 14, 36], and maize [15, 16, 37], key trait-associated loci were identified through GWAS, and the expression patterns and functions of candidate genes were further analyzed using transcriptome sequencing, successfully revealing multiple important genes and their regulatory mechanisms that affect crop yield, quality, and disease resistance. This integrated analysis approach not only improves the accuracy of gene localization but also enhances the understanding of gene function and regulatory networks.

As an important tropical and subtropical fruit, the genetic regulatory mechanisms underlying inflorescence development and fruit set rate in litchi remain incompletely understood. Although previous studies have evaluated external factors, morphological changes, and biochemical mechanisms during the flowering process of litchi, the molecular mechanisms related to the relationship between inflorescence-related traits and fruit set rate still lack thorough investigation. This study aims to comprehensively analyze the complex relationship between inflorescence phenotypic traits and fruit set rate in litchi through the integration of GWAS and transcriptome sequencing, reveal its underlying regulatory mechanisms, and provide a scientific basis for the genetic improvement of litchi.

## Materials and methods

### Litchi germplasm resources

The 219 litchi germplasm resources used in this study were obtained from the National Litchi Germplasm Repository of the Guangdong Academy of Agricultural Sciences, located in Guangzhou City, Guangdong Province, China (geographical coordinates: 23°09′ N and 113°22′ E). The experimental site belongs to a typical marine subtropical monsoon climate zone, with an annual average temperature consistently maintained at approximately 21 °C and an annual precipitation reaching up to 1761.7 mm. The orchard terrain exhibits hilly characteristics, and consistent practices were maintained in soil management and fertilizer application. All 219 selected litchi germplasm resources were mature trees aged between 25 and 30 years, which were in good health, pest- and disease-free, and capable of normal flowering and fruiting every year.

### Determination of inflorescence phenotypic traits

The systematic investigation targeting litchi germplasm resources was officially launched on February 22, 2021, with the core objective of comprehensively assessing the growth status and inherent characteristics of litchi germplasm resources. The investigation process continued until all female flowers were fully open, ensuring comprehensive coverage and high accuracy of the collected data. Meanwhile, given the unique biological characteristics of litchi, this study particularly emphasized completing all data collection before the second round of male flower blooming to avoid potential impacts on the research results from subsequent biological processes. In the selection of research samples, we carefully chose litchi trees located in four different directions: east, south, west, and north, to ensure sample representativeness and diversity. For each direction, we further selected five inflorescences that were of relatively consistent size, in good growth status, and best represented the characteristics of their respective litchi varieties as observation objects. These selected inflorescences exhibited optimal growth status and could therefore fully represent the typical characteristics of their respective varieties. To delve into the characteristics of litchi germplasm resources, this study employed a series of precise measurement indicators for quantitative analysis. Specifically, we first measured the inflorescence length and width of each selected inflorescence to assess its overall morphological characteristics. Secondly, we recorded the number of secondary lateral inflorescences and the number of inflorescence internodes, which effectively reflect the complexity of the inflorescence and its branching structural features. Finally, we also measured the length from the base to the main axis and the length of the fifth last internode, which helped reveal the spatial distribution characteristics and growth dynamics of the inflorescence.

### Measurement of female flower fertilization rate

To monitor the growth dynamics of litchi germplasm resources, this study formulated a detailed recording protocol. From the onset of female flower blooming, the total number of female flowers was recorded every seven days. This recording frequency was deemed capable of accurately reflecting the blooming and fading process of litchi flowers. The recording continued until the ovary developed to the litchi granulation stage (i.e., completion of the fertilization process), ensuring a comprehensive understanding of the reproductive characteristics of litchi germplasm resources.

The FR was calculated using the following formula:

$$\:\text{F}\text{e}\text{m}\text{a}\text{l}\text{e}\:\text{f}\text{l}\text{o}\text{w}\text{e}\text{r}\:\text{f}\text{e}\text{r}\text{t}\text{i}\text{l}\text{i}\text{z}\text{a}\text{t}\text{i}\text{o}\text{n}\:\text{r}\text{a}\text{t}\text{e}\:=\:\frac{\text{n}\text{u}\text{m}\text{b}\text{e}\text{r}\:\text{o}\text{f}\:\text{f}\text{r}\text{u}\text{i}\text{t}\text{i}\text{n}\text{g}}{\text{t}\text{o}\text{t}\text{a}\text{l}\:\text{n}\text{u}\text{m}\text{b}\text{e}\text{r}\:\text{o}\text{f}\:\text{f}\text{e}\text{m}\text{a}\text{l}\text{e}\:\text{f}\text{l}\text{o}\text{w}\text{e}\text{r}\text{s}}\:\times\:\:100\%$$

### Dynamic measurement of fruit set rate

In this study, fruits were screened at stages ranging from ovary development to the bi-lobed stage, with each lobe containing one ovule [38]. Usually, only one ovary can develop into a fruit and the other one atrophies after pollination and fertilization. Five fruit clusters were meticulously selected as research subjects from the east, south, west, and north directions of the litchi trees. These clusters were chosen based on having the highest number of small fruits, the best growth status, and the most optimal overall performance. Subsequently, the total number of small fruits on these selected fruit clusters was recorded every 7 days, and this recording process continued until the 63rd day after flowering, when the seeds were fully mature.

The formula for calculating the weekly fruit set rate is as follows:

$$\:\text{W}\text{e}\text{e}\text{k}\text{l}\text{y}\:\text{f}\text{r}\text{u}\text{i}\text{t}\:\text{s}\text{e}\text{t}\:\text{r}\text{a}\text{t}\text{e}\:=\:\frac{\text{c}\text{u}\text{r}\text{r}\text{e}\text{n}\text{t}\:\text{n}\text{u}\text{m}\text{b}\text{e}\text{r}\:\text{o}\text{f}\:\text{f}\text{r}\text{u}\text{i}\text{t}\text{s}\text{e}\text{t}}{\text{t}\text{o}\text{t}\text{a}\text{l}\:\text{n}\text{u}\text{m}\text{b}\text{e}\text{r}\:\text{o}\text{f}\:\text{f}\text{r}\text{u}\text{i}\text{t}\text{s}\:\text{i}\text{n}\:\text{t}\text{h}\text{e}\:\text{c}\text{o}\text{n}\text{c}\text{a}\text{t}\text{e}\text{n}\text{a}\text{t}\text{i}\text{o}\text{n}\:\text{p}\text{e}\text{r}\text{i}\text{o}\text{d}}\:\times\:\:100\%$$

The formula for calculating the final fruit set rate is as follows:

$$\:\text{F}\text{i}\text{n}\text{a}\text{l}\:\text{f}\text{r}\text{u}\text{i}\text{t}\:\text{s}\text{e}\text{t}\:\text{r}\text{a}\text{t}\text{e}\:=\:\frac{\text{f}\text{i}\text{n}\text{a}\text{l}\:\text{n}\text{u}\text{m}\text{b}\text{e}\text{r}\:\text{o}\text{f}\:\text{f}\text{r}\text{u}\text{i}\text{t}\:\text{s}\text{e}\text{t}}{\text{t}\text{o}\text{t}\text{a}\text{l}\:\text{n}\text{u}\text{m}\text{b}\text{e}\text{r}\:\text{o}\text{f}\:\text{f}\text{r}\text{u}\text{i}\text{t}\text{s}\:\text{i}\text{n}\:\text{t}\text{h}\text{e}\:\text{c}\text{o}\text{n}\text{c}\text{a}\text{t}\text{e}\text{n}\text{a}\text{t}\text{i}\text{o}\text{n}\:\text{p}\text{e}\text{r}\text{i}\text{o}\text{d}}\:\times\:\:100\%$$

### Genotype identification and data analysis in GWAS

Using the filtered SNP data from 219 litchi samples and considering the population structure analysis results as covariates, the FarmCPU model in GAPIT software [22] was employed to conduct genome-wide association analysis for 8 traits. To mitigate false positives arising from multiple testing, the Bonferroni method adjusted for linkage disequilibrium (LD) [39] was used to correct the P-values of the GWAS results. Manhattan plots and Q-Q (Quantile-Quantile) plots were utilized to visualize the association analysis outcomes. The EigenGWAS analyses were conducted using a linear mixed model without covariate matrix in R package (version 3.5.2). When using the mixed linear model (MLM) in TASSEL for association analysis, structure results (Q matrix from admixture analysis), kinship (K matrix), and PCA results were used individually or in combination to correcting for population structure. In the case of the MLMM model for six phenotypic datas of seed trait, we conduct association analysis using both the Bayesian-information and linkage-disequilibrium iteratively nested keyway (BLINK) and fixed and random model circulating probability unification (FarmCPU) algorithms simultaneously with R-package GAPIT version3 (http://www.zzlab.net/GAPIT/GAPIT.library.R). The significance threshold for genome-wide association was determined based on a false discovery rate (FDR-adjusted *p* < 0.05). The raw sequencing data for GWAS analysis in this study have been uploaded to the NCBI Sequence Read Archive (SRA) database, with the BioProject accession number PRJNA1107979.

### Transcriptome sequencing and differential gene expression analysis

Library construction and sequencing were conducted by Guangzhou Genedenovo Biotechnology Co., Ltd. Two typical litchi varieties, Edanli (with large and sparse inflorescences) and Houxian (with small and dense inflorescences), were selected for sequencing at flowering, 10 days after flowering, and 20 days after flowering, designated as S1, S2, and S3, respectively. FeatureCounts was used to count gene expression levels from quality-controlled data. It maps sequencing data to a reference genome or transcriptome assembly and calculates count values for each gene or transcript. Differential gene expression analysis was performed using the DESeq2 package [40] in R software, with the threshold for significant differential expression set at FDR (False Discovery Rate) < 0.05 and|log2(Fold Change)| > 2. Visualization was conducted using the ggplot2 package in R software [41]. To explore the functions of differentially expressed genes, GO (Gene Ontology) and KEGG (Kyoto Encyclopedia of Genes and Genomes) enrichment analyses were performed using Omicshare tools to predict gene functions. GO and KEGG pathway enrichment analyses and visualization were conducted for differentially expressed genes, and GO terms and KEGG pathways were considered significantly enriched when *p* < 0.05. The raw sequence data reported in this paper have been deposited in the Genome Sequence Archive (Genomics, Proteomics & Bioinformatics 2021) in National Genomics Data Center (Nucleic Acids Res 2022), China National Center for Bioinformation/Beijing Institute of Genomics, Chinese Academy of Sciences (GSA: CRA026095) that are publicly accessible at https://ngdc.cncb.ac.cn/gsa.

### qRT-PCR expression analysis of candidate genes

Total RNA was extracted using the RNAprep Pure Kit (Polysaccharides & Polyphenolics-rich, TIANGEN). cDNA synthesis was performed using the PrimeScriptTM RT reagent Kit with gDNA Eraser (Perfect Real Time, TakaRa). qRT-PCR was conducted in a CFX96 real-time PCR system (Bio-Rad, CA) using iTaq Universal SYBR Green Supermix (Bio-Rad). Primers are shown in Table S1.

### Data processing and statistical analysis

Phenotypic traits were analyzed and plotted using Origin 2022. Statistical analysis was performed using SPSS software (SPSS 27.0, SPSS Inc.). For statistical analysis, one-way ANOVA was conducted. The significance levels for ANOVA were set at *p* = 0.05 (significant) or *0.01* (highly significant).

## Results and analysis

### Descriptive statistics and variability analysis

The variation in the coefficient of variation (CV) reveals the inherent characteristics of genetic diversity and individual differences, providing us with deep insights into the specific aspects of genetic diversity. When a trait exhibits a high CV, it typically indicates a rich genetic background underlying that trait, making it easier to identify varieties. As evident from the results in Table 1, the CV of NFFI is the highest, reaching 61.99%, suggesting that NFFI is a particularly significant trait in distinguishing differences among the 219 litchi varieties analyzed in this study. In contrast, the CVs of NII and NL are relatively low, being 23.87% and 23.13% respectively, hinting at their better genetic stability. Further observation of the standard deviation data in Table 1 reveals that the standard deviations of BMAL and I5IL are less than 1.5, while those of other traits are all greater than 2.0. This result indicates that the data for BMAL and I5IL are more concentrated compared to other traits, which is also supported by the range data in the table. When exploring the genetic diversity of phenotypic traits related to litchi inflorescences, we introduced the Simpson index and Shannon Weaver index. As evident from the data in Table 2, the Simpson indices of the eight inflorescence-related traits range from 0.9937 to 0.9952, while the Shannon Weaver indices range from 7.5739 to 7.7726. The ranges of these two indices both indicate that litchi resources exhibit high genetic diversity in inflorescence-related phenotypic traits.

**Table 1:** Analysis of phenotypic traits variation related inflorescence in 219 litchi resources

Trait	IL cm	IW cm	NSLI	NII	BMAL	I5IL	NFFI	FR %
Min.	14.40	8.20	5.00	4.00	0.70	0.80	12.00	4.27
Max.	45.70	39.00	22.00	16.00	10.10	8.80	445.00	91.09
Range	31.30	30.80	17.00	12.00	9.40	8.00	433.00	86.82
Mean	25.15	17.27	13.48	9.10	3.00	2.55	98.93	45.43
Standard difference	5.82	5.00	3.40	2.17	1.34	0.96	61.33	21.75
Coefficient of variation (CV; %)	23.13	28.94	25.23	23.87	44.75	37.65	61.99	47.87

**Table 2:** Analysis of genetic diversity index for traits related inflorescence phenotypic in 219 litchi resources

Trait	Simpson diversity index	Shannon-Weaver diversity index
IL (cm)	0.9952	7.7726
IW (cm)	0.995	7.7532
NSLI	0.9951	7.7635
NII	0.9952	7.7702
BMAL	0.9945	7.68
I5IL	0.9948	7.7195
NFFI	0.9937	7.5739
FR(%)	0.9944	7.6301

### Correlation between fruit set rate and related agronomic traits

**Fig. 1:**
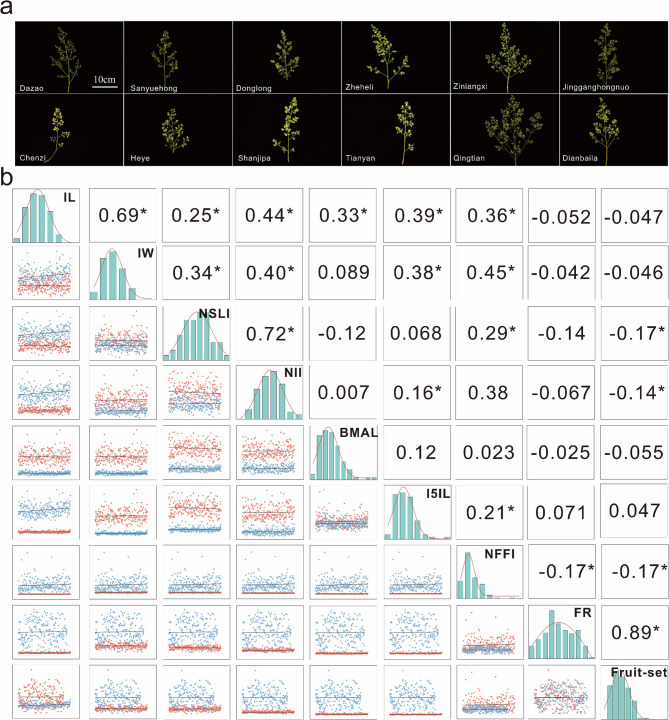
Phenotypic traits of inflorescences in different litchi varieties and their correlation with fruit set rate. Phenotypic photos of inflorescence traits in different litchi varieties (**a**) and their correlation analysis (**b**)

In this study, significant differences in inflorescence shapes were observed among different litchi varieties (Fig. 1a), which had a notable impact on the fruit set rate of litchi. This is because the same inflorescence of litchi undergoes three openings in the sequence of male-female-male, with a relatively long flowering period (about 20 days) and a large number of flowers. Therefore, a substantial amount of energy and nutrients are consumed during flowering, affecting later fertilization and fruit set, and ultimately influencing the final yield. In production, the flowering and fruiting of litchi are described as “flowering without fruiting”, “one fruit from thousand flowers”, and “cherishing flowers at the expense of fruits”. Furthermore, the size of the litchi inflorescence (litchi has cymose inflorescences arranged in a panicle-like structure) also affects the final yield. Typically, long and large (sparse) inflorescences have a lower fruit set rate, while short, sturdy, and compact inflorescences have a higher fruit set rate. This study selected the fruit set rate of different litchi varieties in the first week and conducted a correlation analysis with the aforementioned typical agronomic traits (Fig. 1b). The analysis results indicated a significant positive correlation between the fruit set rate and the fertilization rate, while there were significant negative correlations with NSLI, NII, and NFFI (*p < 0.05*). Moreover, all these traits maintained significant correlations with the fruit set rate at each stage (Figure S1). Additionally, we performed histogram analysis on these eight typical traits, and the results showed that they all exhibited obvious normal distribution characteristics. The histogram of lychee phenotypic traits in 2020 also demonstrates this result (Figure S2, Table S2). This indicates that the agronomic trait data related to the fruit set rate of different litchi varieties collected in this study have high reliability and stability.

### Comprehensive analysis of fruit set rate

**Fig. 2:**
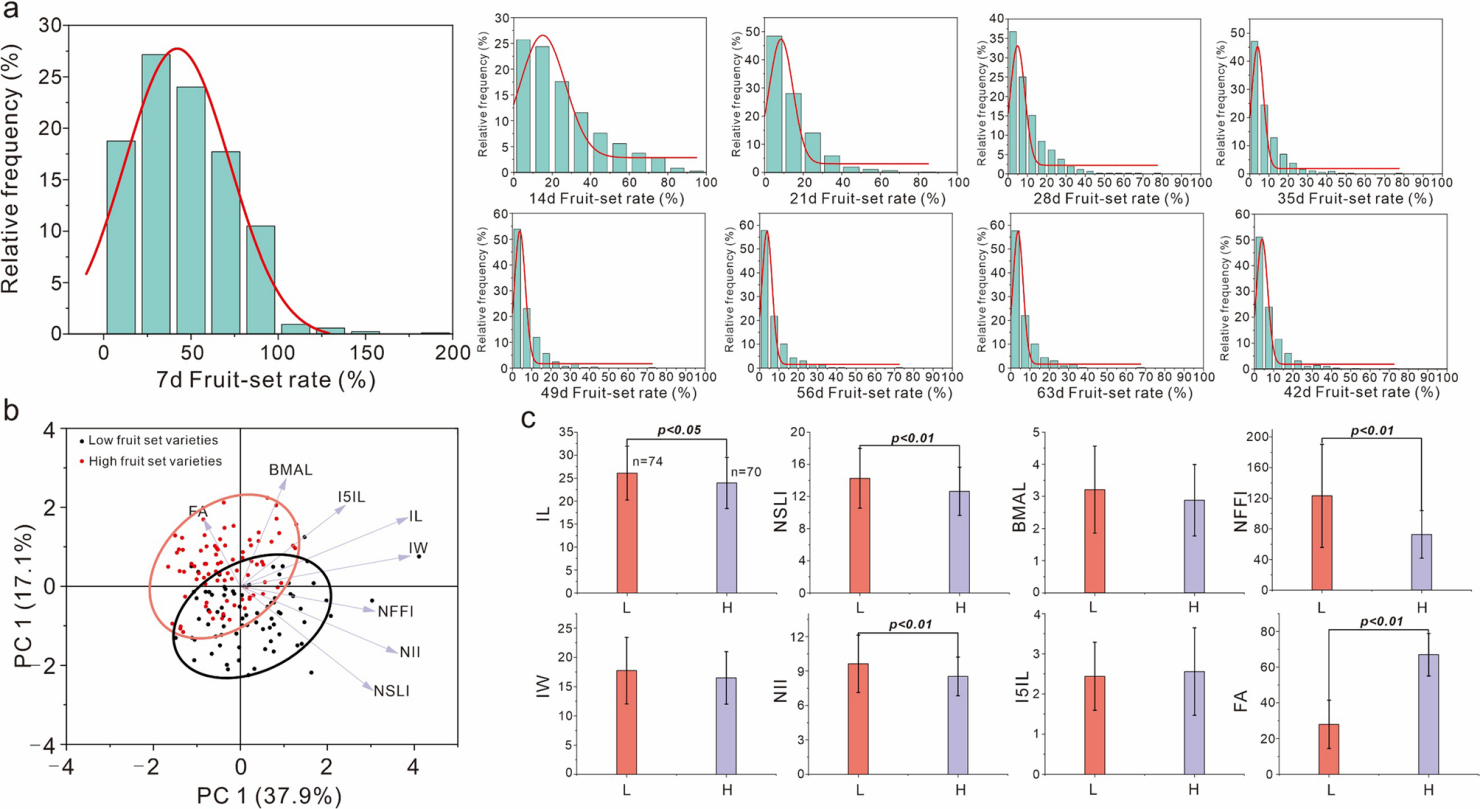
Comprehensive analysis of the dynamic changes in inflorescence traits and fruit set rate of litchi. **a **Fruit set rates of 219 litchi germplasm resources at 7, 14, 21, 28, 42, 49, 56, and 63 days after flowering; **b **PCA analysis of eight typical traits in 70 high fruit set rate varieties and 74 low fruit set rate varieties; **c **Differential analysis of eight typical traits between 70 high fruit set rate varieties and 74 low fruit set rate varieties

Upon detailed observation and analysis of the histograms, it was evident that the fruit set rate of litchi exhibited the most prominent normal distribution characteristics in the first week. However, as time progressed from the second to the ninth week, the distribution gradually shifted towards a skewed normal distribution (Fig. 2a). This change clearly indicated a decreasing trend in the fruit set rate of litchi over time. To further explore the underlying patterns of litchi fruit set rate, we classified 219 litchi varieties into high fruit set rate varieties (with a fruit set rate above 70%, totaling 70 varieties) and low fruit set rate varieties (with a fruit set rate below 30%, totaling 74 varieties) based on their fruit set rates. By effectively applying the mathematical dimension reduction method of Principal Component Analysis (PCA), we found that these two types of litchi varieties exhibited distinct separation in the PCA results (Fig. 2b). This finding not only aligned with the previous correlation analysis results, where high fruit set rate varieties were often accompanied by lower NSLI, NII, and NFFI values, but also provided new perspectives and entry points for our subsequent research. Based on this, we further conducted a detailed comparative analysis of eight typical traits between high and low fruit set rate varieties. The results showed significant differences (*p < 0.05*) in five traits: IL, NSLI, NFFI, NII, and FA, between high and low fruit set rate litchi varieties. Specifically, compared with high fruit set rate varieties, low fruit set rate varieties exhibited increases of 8.23%, 11.36%, 40.94%, and 11.41% in IL, NSLI, NFFI, and NII, respectively, while FA decreased by 139.18% (Fig. 2c). This finding not only revealed significant differences in trait levels between high and low fruit set rate varieties but also provided important theoretical basis and data support for our further exploration of the regulatory mechanisms of litchi fruit set rate.

### GWAS of agronomic traits and candidate genes related to fruit set rate

**Fig. 3:**
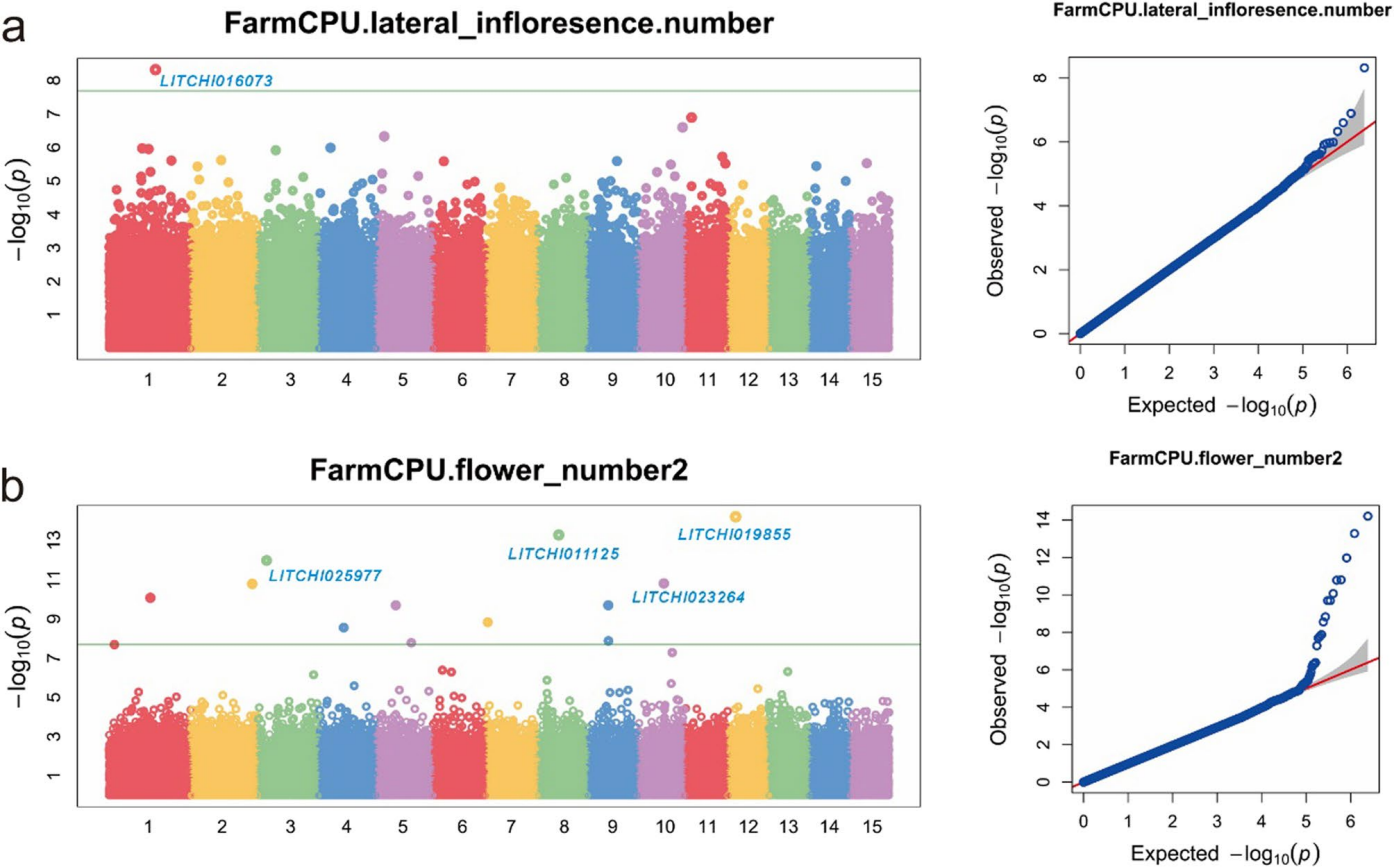
Manhattan plot and QQ plot of genes associated with key SNP loci identified through genome-wide association analysis for NSLI (**a**) and NFFI (**b**)

To precisely identify SNP loci significantly associated with target traits, this study employed the FarmCPU algorithm to conduct genome-wide association analysis on multiple trait datasets. Among the 8 agronomic traits related to fruit set rate, only two traits, NSLI and NFFI, detected significant SNP loci (Fig. 3), while other traits (IL, IW, NII, BMAL and I5IL) were not associated with significant SNPs (Figure S3). Specifically, one significant SNP locus was detected for the NSLI trait, while twelve significant SNP loci were detected for the NFFI trait (Table 3). Based on these significant SNP loci, we further searched for candidate genes related to inflorescence development within a 200 kb region upstream and downstream of each locus. The results showed that the SNP locus significantly associated with NSLI was located on chromosome 1, encoding the main product of UBP1-associated proteins. For NFFI, the four significantly associated SNP loci were located on chromosomes 12, 8, 3, and 10, respectively, encoding Solute carrier family, Exocyst complex component SEC3A, Acid phosphatase, and Enolase (Table 3).

**Table 3:** Information on genes significantly associated with NSLI and NFFI

Traits	Gene	Chromosome	Allelic genes	Position (BP)	*p* value	Gene annotation or coding proteins
NSLI	LITCHI016073	1	T: C	28,261,616	4.78E-09	UBP1-associated proteins
NFFI	LITCHI019855	12	T: C	4,691,791	6.26E-15	Solute carrier family 40 member 1
	LITCHI011125	8	C: T	12,866,931	5.30E-14	Exocyst complex component SEC3A
	LITCHI025977	3	C: T	5,533,862	1.06E-12	Acid phosphatase
	LITCHI023264	10	C: T	15,764,266	1.57E-11	Enolase

### Transcriptome analysis

**Fig. 4:**
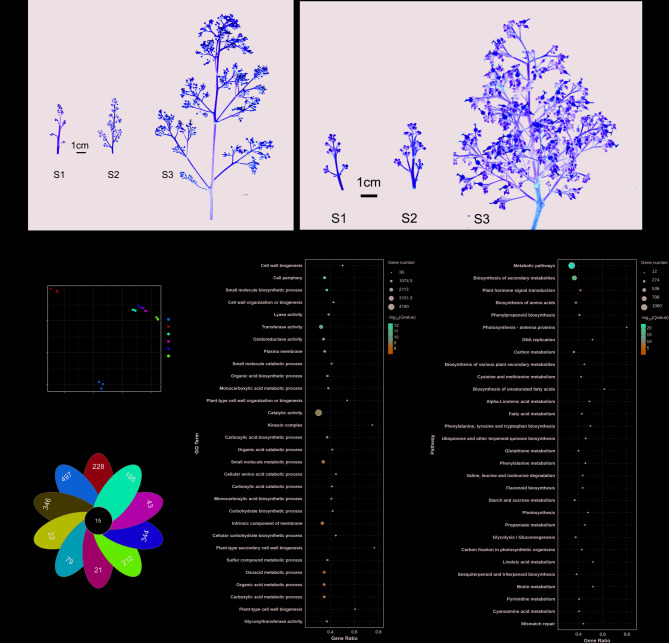
Transcriptome analysis to verify the role of key genes in the formation of inflorescence traits. **a **Sparse inflorescences in Edanli (left) and dense inflorescences in Houxian (right); **b **PCA analysis of transcriptome during the S1 stage; **c **Analysis of differential gene expression in the transcriptome during the S1 stage. **d **GO enrichment of expressed genes during the S1 stage; (ee) KEGG enrichment of expressed genes during the S1 stage

After identifying candidate genes significantly associated with litchi inflorescence traits through GWAS, this study further verified these results using transcriptome analysis. Specifically, two litchi varieties with distinct inflorescence traits, Edanli (characterized by sparse inflorescences) and Houxian (characterized by dense inflorescences), were selected as study subjects (Fig. 4a). The comparison of the eight trait data of these two varieties can be seen in Table S5. Transcriptome analysis was conducted at three key developmental stages for these two varieties, starting from flowering, 10 days after flowering, and 20 days after flowering, corresponding to stages S1, S2, and S3, respectively. PCA results showed that the gene expression patterns of these two typical litchi varieties at different developmental stages could be significantly distinguished, indicating significant differences in gene expression between them (Fig. 4b). Venn diagram analysis further revealed a large number of differentially expressed genes (DEGs) between the two varieties at different developmental stages (Fig. 4c). Based on GO classification statistics, we found that the gene expression differences between Edanli and Houxian were mainly concentrated in the molecular function category. Specifically, through the secondary classification of GO terms, we found that these differences were mainly annotated in catalytic activity and transferase activity (Fig. 4d). Furthermore, KEGG pathway enrichment analysis revealed that the two typical litchi varieties mainly affected metabolic pathways and biosynthesis of secondary metabolites during the three developmental stages (Fig. 4e). GO enrichment analysis and KEGG enrichment analysis at S2 and S3 stages also supported this result (Figure S4). Combining the results of GWAS, we identified molecules such as UBP1-associated proteins, Solute carrier family 40 member (SLC40), Exocyst complex component SEC3A, Acid phosphatase, and Enolase in plants, which affect metabolic pathways and the synthesis of secondary metabolites through different mechanisms. Specifically, these molecules collectively maintain normal plant growth and inflorescence development by regulating protein stability, metal ion transport, vesicular transport, phosphate cycling, and the glycolytic pathway [42–45]. Furthermore, according to the results of transcriptome analysis, we found significant differential expression of these five key genes—UBP1-associated proteins, SLC40, SEC3A, Acid phosphatase, and Enolase—between the two litchi varieties (Figure S5). Therefore, the results of transcriptome analysis further confirmed the important role of the key genes identified by GWAS in the formation of litchi inflorescence traits.

### qRT-PCR validation

To further validate the aforementioned results, this study employed qRT-PCR technology to quantitatively analyze the expression levels of the target genes. The analysis revealed that all five genes examined exhibited significant differential expression patterns. Specifically, for the gene *LITCHI016073* identified by NSLI, the expression level of *LITCHI016073* in the Houxian was significantly higher than that in the Edanli. UBP1-associated proteins can elicit reproductive-related responses such as flowering and fruiting through gibberellin signaling [46], which contributes to the denser inflorescences in the Houxian. Among the four genes identified by NFFI, the Edanli showed significantly higher expression of the gene *LITCHI019855* compared to the Houxian. High expression of the Solute carrier family may lead to a reduction in the number of female flowers [47], resulting in sparse inflorescences in the Edanli. Conversely, the Houxian exhibited significantly higher expression levels of three genes, *LITCHI011125*, *LITCHI025977*, and *LITCHI023264*, compared to the Edanli. This is primarily because these three genes significantly promote the development of female flowers, thereby enhancing the development of litchi inflorescences and female flowers [42–45], which could be the reason for the denser inflorescences in the Houxian.

## Discussion

**Fig. 5:**
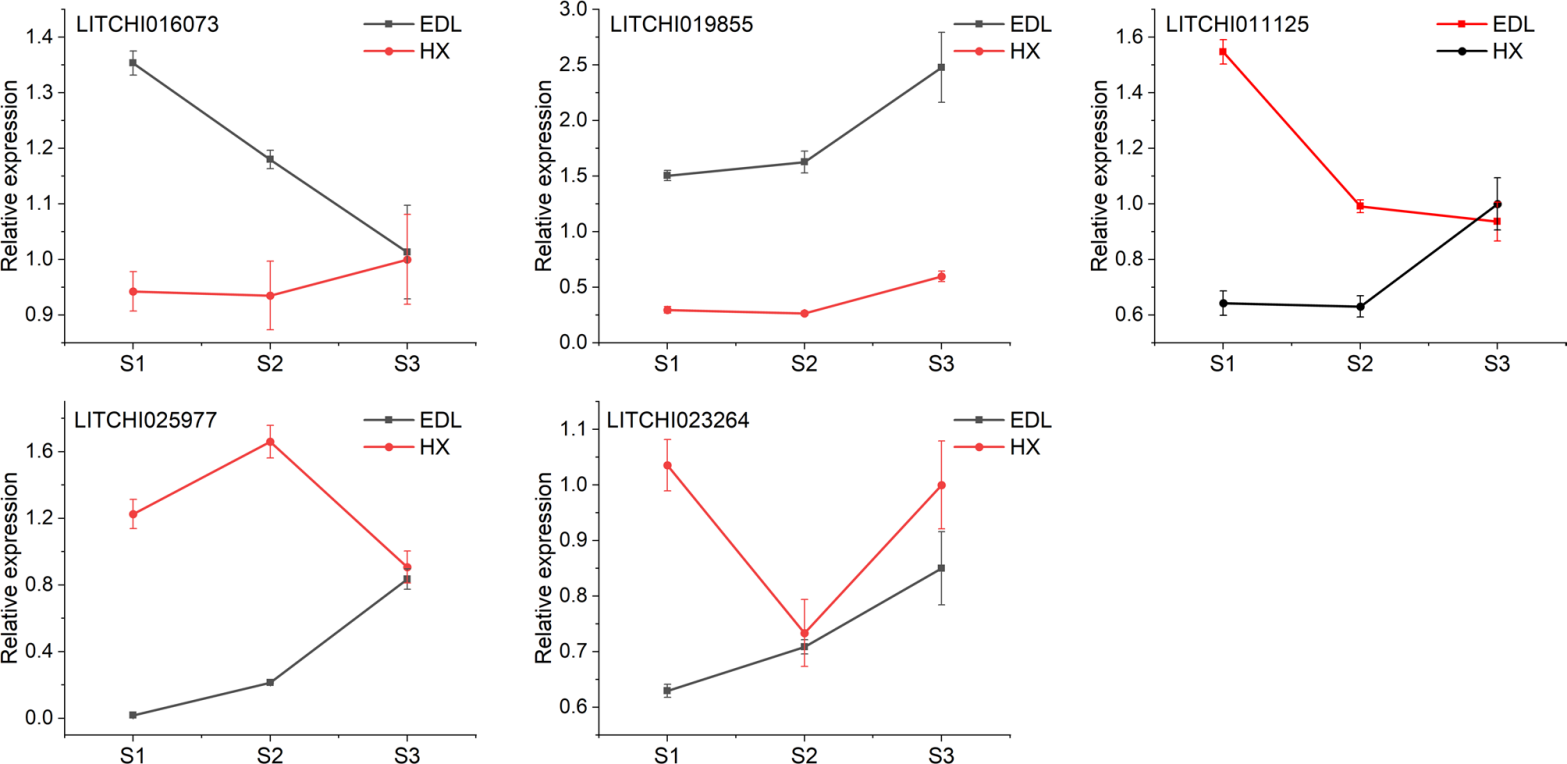
Verification of expression differences in candidate genes using qRT-PCRVerification of expression differences in candidate genes using qRT-PCR

Enhancing the fruit set rate of litchi trees is of paramount importance for the development of the litchi industry. As a significant tropical and subtropical fruit, the yield of litchi directly impacts the economic returns of fruit farmers and the stability of market supply. However, litchi production is constrained by multiple factors, among which the low fruit set rate is a prevalent issue worldwide [48–50]. Initially, PCA and multiple linear regression analysis in this study revealed that the fruit set rate of litchi is highly influenced by the phenotypic traits of inflorescences (Figs. 1 and 2, S6, Table S3, S4). Although previous research has suggested agronomic practices such as pruning main and lateral branches to improve the fruit set rate [51, 52], the underlying reasons have not been further elucidated. Therefore, optimizing the inflorescence structure and female flower development to enhance the fruit set rate can significantly increase the number of fruits per litchi tree, thereby boosting overall yield and providing higher economic returns for fruit farmers. This study used 219 litchi germplasm resources as experimental subjects to investigate the dynamic changes of inflorescence phenotypes during fruit development. GWAS can identify SNP loci associated with specific phenotypes across the whole genome, but due to linkage disequilibrium, the regions they locate are often large and contain multiple genes [53, 54]. Transcriptomics, on the other hand, provides information on gene expression profiles at different developmental stages or conditions, helping to screen candidate genes actually involved in trait regulation [17, 55]. By combining GWAS and transcriptomics analysis, we could further narrow down the range of candidate genes within the associated regions, thereby improving the accuracy of gene mapping. GWAS results showed significant SNP loci detected for the two traits, NSLI and NFFI. Further analysis of the 200 kb upstream and downstream regions of these loci, combined with transcriptome gene expression data, revealed candidate genes closely related to inflorescence development (Fig. 3; Table 1). Specifically, the significant SNP locus associated with the NSLI trait encodes UBP1-associated proteins, which may regulate the development of secondary lateral inflorescences by influencing gibberellin signaling and affecting the reproduction and flowering of inflorescences [46]. For the NFFI trait, we detected multiple significant SNP loci located on different chromosomes, encoding various key proteins including solute carrier family 40 members (SLC40), exocyst complex component SEC3A, acid phosphatase, and enolase (Fig. 3; Table 1). These proteins play important roles in metabolic pathways, metal ion transport, vesicular transport, and phosphate cycling, jointly affecting the development and number of female flowers in litchi [42–45]. Transcriptome analysis further validated the GWAS results. By selecting litchi varieties with typical inflorescence traits, Edanli and Houxian, and performing transcriptome sequencing at different developmental stages, we found significant differences in gene expression between these two varieties. PCA results showed that their gene expression patterns at different developmental stages could be significantly distinguished. Venn diagram analysis revealed a large number of differentially expressed genes, which were mainly enriched in molecular function categories such as catalytic activity and transferase activity. KEGG pathway enrichment analysis indicated that these differential genes mainly affected metabolic pathways and secondary metabolite synthesis, consistent with the functions of key genes mapped by GWAS. Notably, key genes such as UBP1-associated proteins, SLC40, SEC3A, acid phosphatase, and enolase showed significant differences in expression levels between the two litchi varieties, further confirming their important roles in inflorescence trait formation and fruit set rate regulation (Fig. 5, S5).

GWAS combined with transcriptome analysis has demonstrated significant scientific significance and practical value in the localization of genes related to inflorescence traits and fruit set rate in litchi. The genes identified in this study play crucial roles in the development of litchi inflorescences and the regulation of fruit set rate. These genes, as potential targets for genetic improvement, can be targeted for modification through gene editing or transgenic technology, offering the prospect of developing new litchi varieties with improved inflorescence traits and higher fruit set rates. For instance, by upregulating the expression of genes promoting female flower development or downregulating the expression of genes inhibiting female flower development, the inflorescences can become more compact, significantly enhancing the fruit set rate of litchi and ultimately increasing its yield. The discovery of these genes has deepened our understanding of the molecular mechanisms underlying litchi inflorescence development and provided a theoretical basis for optimizing existing breeding strategies and developing new breeding techniques. Furthermore, the research on these genes and their regulatory mechanisms may also offer insights and references for the genetic improvement of other fruit tree crops. The value of these genes has been further validated in practical applications. For example, qRT-PCR experiments revealed significant differences in the expression patterns of these genes among different litchi varieties (Fig. 5), and these differences were closely associated with litchi inflorescence traits and fruit set rate. These experimental results not only support the reliability of the GWAS and transcriptome screening results but also provide important clues for subsequent gene function verification and breeding practices.

## Conclusion

This study conducted a comprehensive analysis of 219 litchi germplasm resources to investigate the relationship between inflorescence-related phenotypic traits and fruit set rate. Through quantitative analysis of eight key agronomic traits, we uncovered significant correlations between fruit set rate and inflorescence traits such as the number of secondary lateral inflorescences, the number of inflorescence internodes, and the number of female flowers per inflorescence. GWAS analysis identified significant SNP loci associated with two inflorescence traits, NSLI and NFFI, revealing potential candidate genes. Joint analysis of GWAS and transcriptomics data successfully narrowed down the range of candidate genes, identifying key regulators such as UBP1-related protein, SLC40, SEC3A, acid phosphatase, and enolase. These genes play crucial roles in inflorescence development and fruit set rate regulation by modulating processes including protein stability, metal ion transport, vesicular transport, phosphate cycling, and glycolytic pathways. Transcriptome analysis further validated the GWAS results, confirming the expression patterns and functions of these key genes in litchi inflorescence and fruit development.

## Supplementary Information

Supplementary Material 1.

## Data Availability

Data is provided within the manuscript or supplementary information files.

## Abbreviations

IL: Inflorescence length
IW: Inflorescence width
NSLI: Number of secondary lateral inflorescences
NII: Number of inflorescence internodes
BMAL: Base to main axis length
I5IL: Inverted 5th internode length
NFFI: Number of female flowers per inflorescence
FR: Fertilization rate
