# Supplementary material for: GWAS combined with transcriptomics revealed key regulatory genes for inflorescence traits and fruit set rate in Litchi (Litchi chinensis Sonn.)
Source: BMC Genomics. 2025 Jul 26;26:692. doi: 10.1186/s12864-025-11796-3 (PMC12297726; doi:10.1186/s12864-025-11796-3)
Supplement: Supplementary file 1 — Supplementary Material 1. [file 12864_2025_11796_MOESM1_ESM.docx]

**Table S1 qRT-PCR Primer Information**

| **Primer** | **Primer sequence (5'to3')** | **Base number** | **Purification method** | **OD** |
| --- | --- | --- | --- | --- |
| qLc025977-F | TCTCGGGTGAGACAAGGTCT | 20 | PAGE | 2 |
| qLc025977-R | AAGTCCCTCAATGCTGCTCC | 20 | PAGE | 2 |
| qLc023264-F | GGGGGAAATCATGCTGGGAA | 20 | PAGE | 2 |
| qLc023264-R | TAGACTTCACTGCCCATGCG | 20 | PAGE | 2 |
| qLc019855-F | CTGGGGCTGTGGATGTTTGA | 20 | PAGE | 2 |
| qLc019855-R | AATGTAAGCCGCCAAGTCCA | 20 | PAGE | 2 |
| qLc016073-F | CAGCGATTGTGATGGAGGGT | 20 | PAGE | 2 |
| qLc016073-R | TCATGTTGGTGGCAGTGGTT | 20 | PAGE | 2 |
| qLc011125-F | AGTGTTGCACCTCCTCAGTG | 20 | PAGE | 2 |
| qLc011125-R | GCCAAGCACATCTTTGCACA | 20 | PAGE | 2 |

Table S2 Multiple linear regression analysis results of 8 inflorescence related traits and fruit setting-rates of different time periods.

| **Different time periods fruit-set rate** | **R^2^** | **F value** | **significant** |
| --- | --- | --- | --- |
| 7-day | 0.8073 | 100.52 | 0.00 |
| 14-day | 0.4775 | 21.93 | 0.00 |
| 21-day | 0.3269 | 11.65 | 0.00 |
| 28-day | 0.3026 | 10.41 | 0.00 |
| 35-day | 0.2734 | 9.03 | 0.00 |
| 42-day | 0.2621 | 8.53 | 0.00 |
| 49-day | 0.2551 | 8.22 | 0.00 |
| 56-day | 0.2490 | 7.96 | 0.00 |
| 63-day | 0.2346 | 7.36 | 0.00 |

Table S3 Factor loading of the PCA for 219 litchi resources

| **Trait** | **Principal component** | | |
| --- | --- | --- | --- |
|  | **PC1** | **PC2** | **PC3** |
| IL | 0.775 | 0.383 | -0.007 |
| IW | 0.808 | 0.206 | 0.075 |
| NSLI | 0.636 | -0.603 | 0.099 |
| NII | 0.743 | -0.421 | 0.135 |
| BMAL | 0.161 | 0.648 | -0.335 |
| I5IL | 0.484 | 0.442 | 0.254 |
| NFFI | 0.641 | -0.070 | -0.193 |
| FR | -0.186 | 0.222 | 0.880 |
| variance (%) | 36.44% | 17.48% | 12.79% |
| Accumulated variance (%) | 36.44% | 53.92% | 66.71% |

**NOTE**: IL: Inflorescence Length; IW: Inflorescence Width; NSLI: Number of Secondary Lateral Inflorescences; NII: Number of Inflorescence Internodes; BMAL: Base to Main Axis Length; I5IL: Inverted 5th Internode Length; NFFI: Number of Female Flowers per Inflorescence; FR: Fertilization Rate

Table S4 The correlation coefficients between 8 inflorescence related traits with F value

| Trait | Correlation coefficients |
| --- | --- |
| IL | 0.838** |
| IW | 0.830** |
| NSLI | 0.425** |
| NII | 0.588** |
| BMAL | 0.285** |
| I5IL | 0.637** |
| NFFI | 0.538** |
| FR | 0.066 |

**NOTE**: IL: Inflorescence Length; IW: Inflorescence Width; NSLI: Number of Secondary Lateral Inflorescences; NII: Number of Inflorescence Internodes; BMAL: Base to Main Axis Length; I5IL: Inverted 5th Internode Length; NFFI: Number of Female Flowers per Inflorescence; FR: Fertilization Rate

**Table S5 Comparison of Eight Trait Data Related to Fruit Set Rate between Houxian and Edanli**

|  | **IL** | **IW** | **NSLI** | **NII** | **BMAL** | **I5IL** | **NFFI** | **FR** |
| --- | --- | --- | --- | --- | --- | --- | --- | --- |
| Houxian | 18.33  ±1.42 | 13.43  ±1.15 | 13.33  ±2.52 | 8  ±1.0 | 11.73  ±1.44* | 3.83  ±0.35 | 99.67  ±2.08 | 50.42  ±1.80* |
|  |  |  |  |  |  |  |  |  |
| Edanli | 32.03  ±1.82** | 25.83  ±1.16** | 16.67  ±1.53 | 10.33  ±1.53 | 6.30  ±0.22 | 3.75  ±0.12 | 211  ±10.54* | 41.98  ±2.32 |

Note: *represents significant difference, and ** represents extremely significant difference. IL: Inflorescence Length; IW: Inflorescence Width; NSLI: Number of Secondary Lateral Inflorescences; NII: Number of Inflorescence Internodes; BMAL: Base to Main Axis Length; I5IL: Inverted 5th Internode Length; NFFI: Number of Female Flowers per Inflorescence; FR: Fertilization Rate


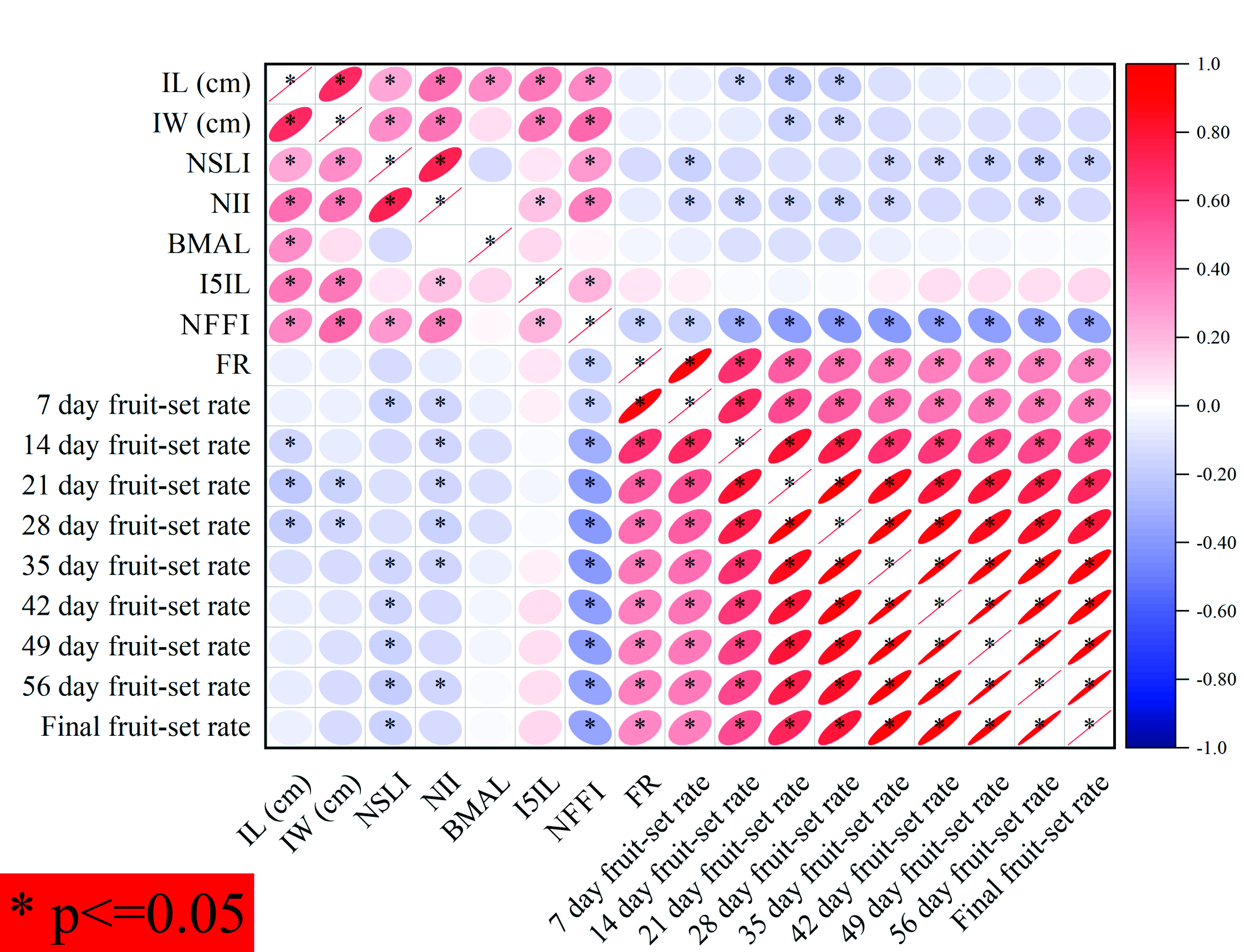


**Figure S1 Correlation Analysis of Fruit Set Rate and Inflorescence Traits Across Nine Stages**


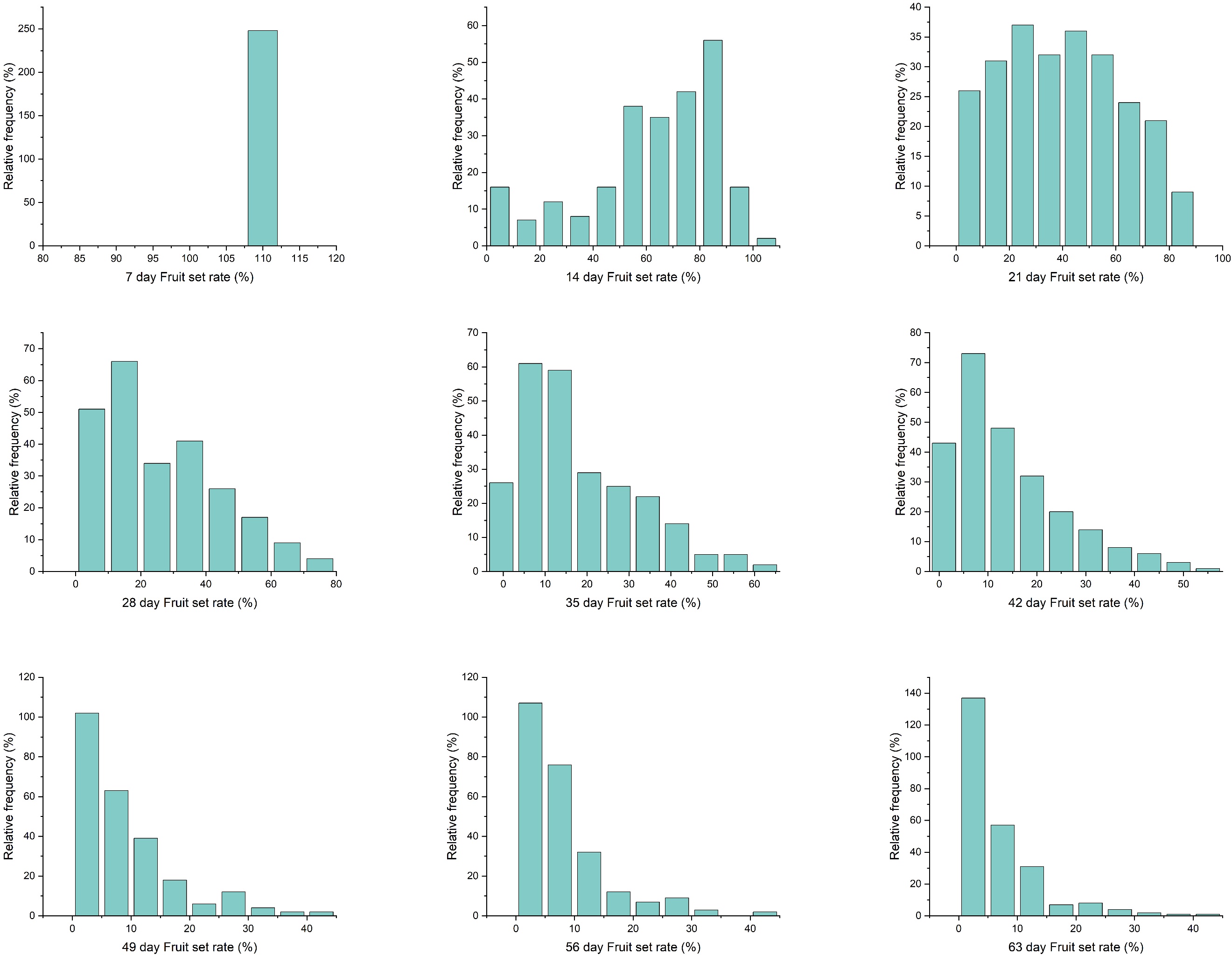


**Figure S2 Dynamic changes in litchi fruit setting rate in 2020**


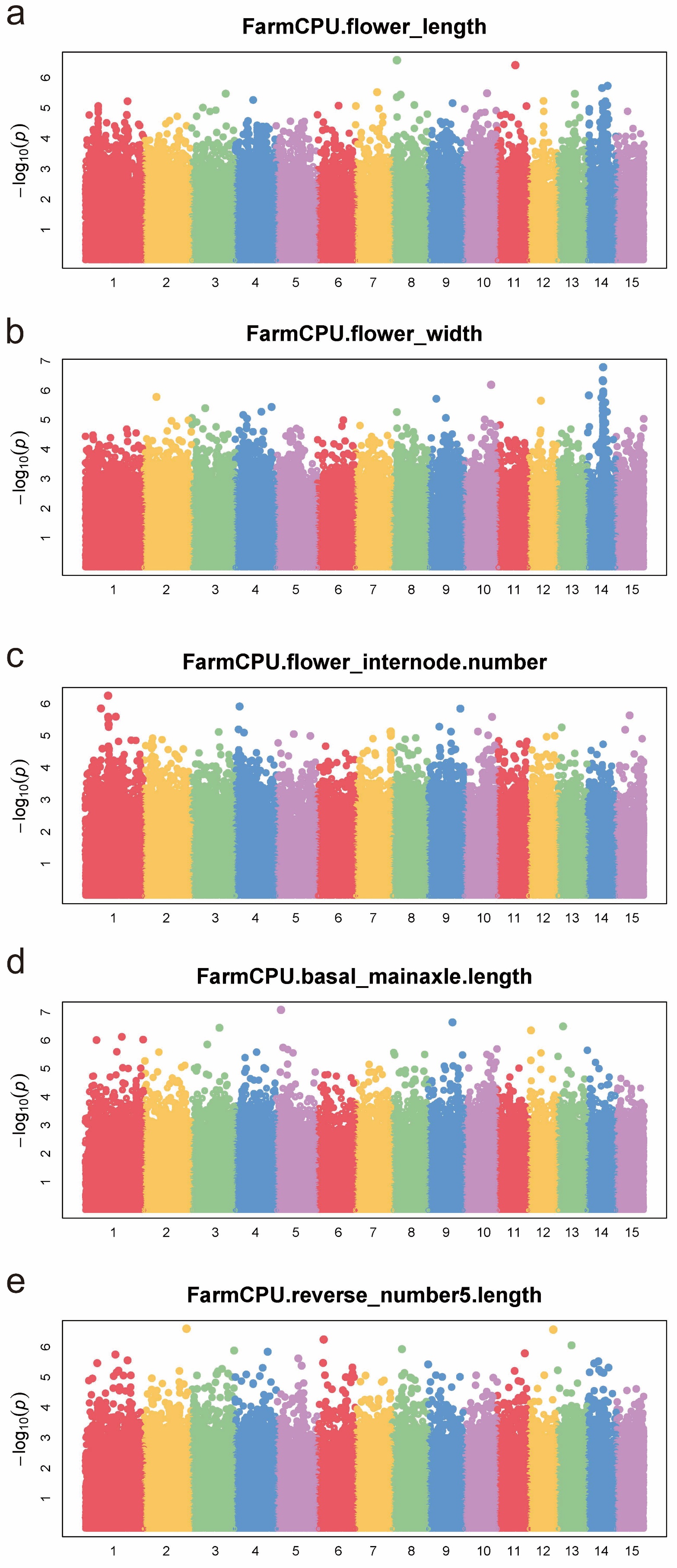


**Figure S3 Manhattan plot of genome-wide association analysis revealing key SNP loci associated with IL(a), LW(b), NII(c), BMAL(d), and I5IL(d)**


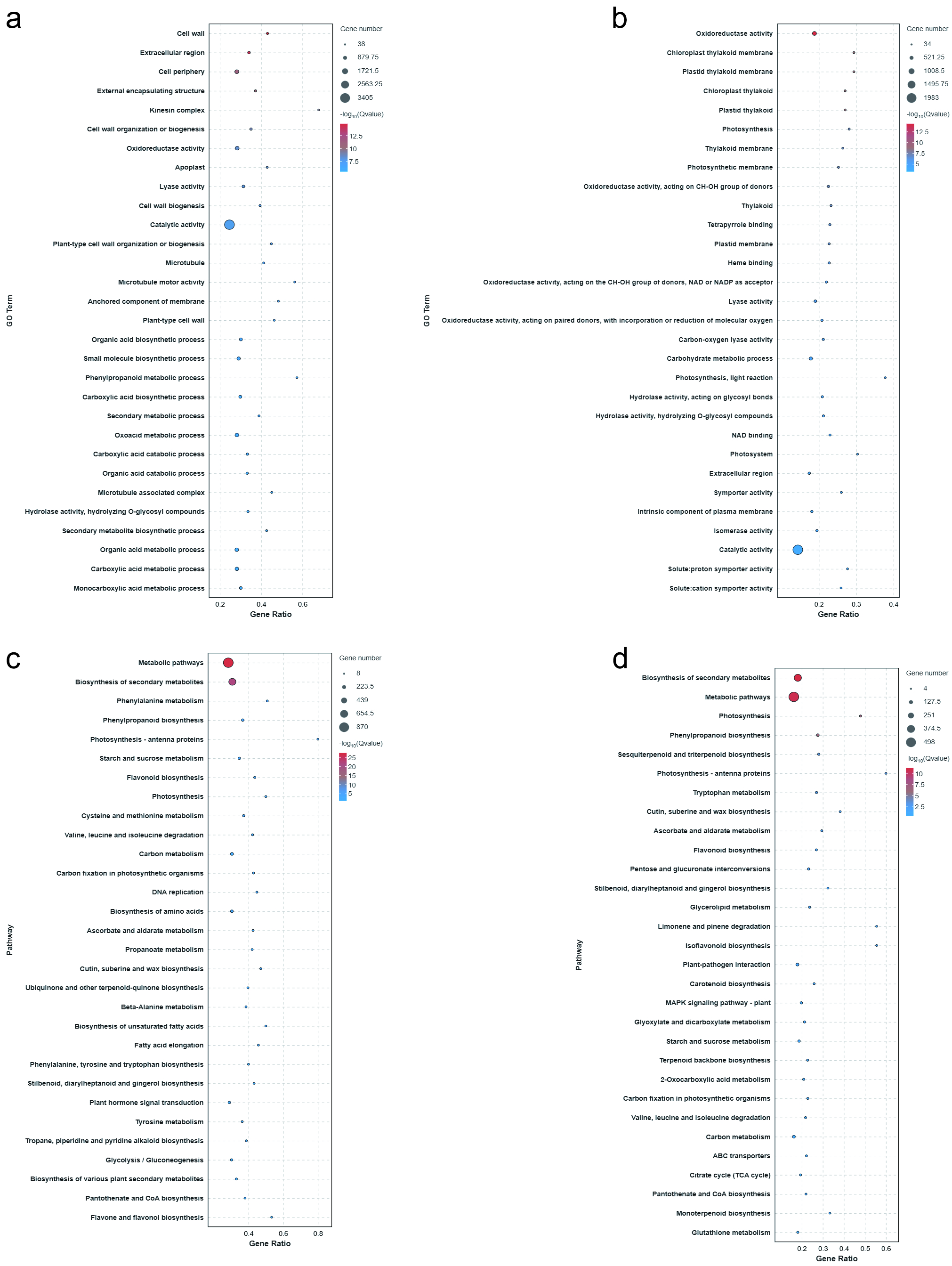
 **Figure S4 GO (a, b) and KEGG (c, d) enrichment analyses of genes expressed during S2 and S3 stages**

**
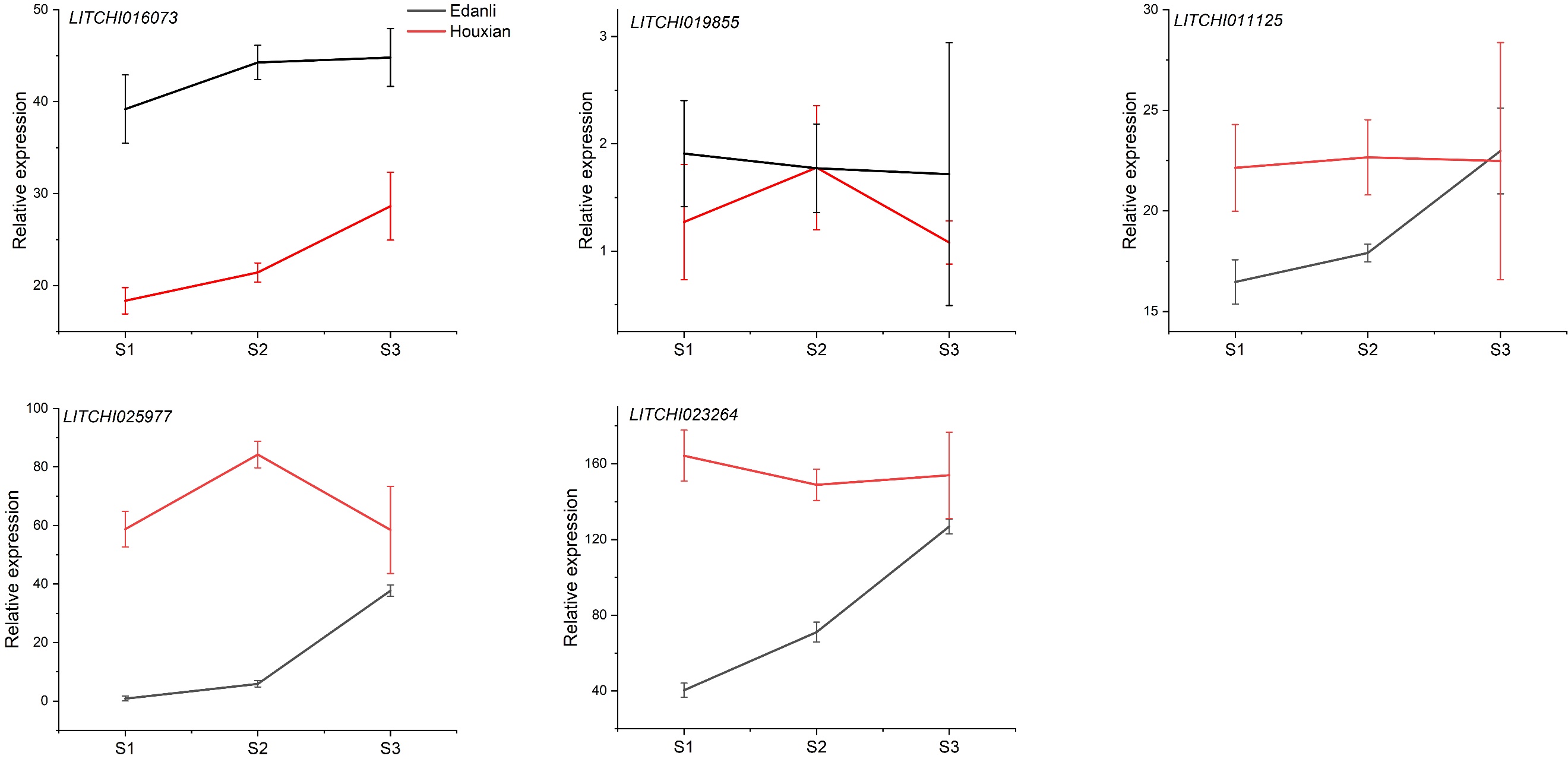
**

**Figure S5 Expression levels of five key genes during the S1, S2 and S3 stages by transcriptome**


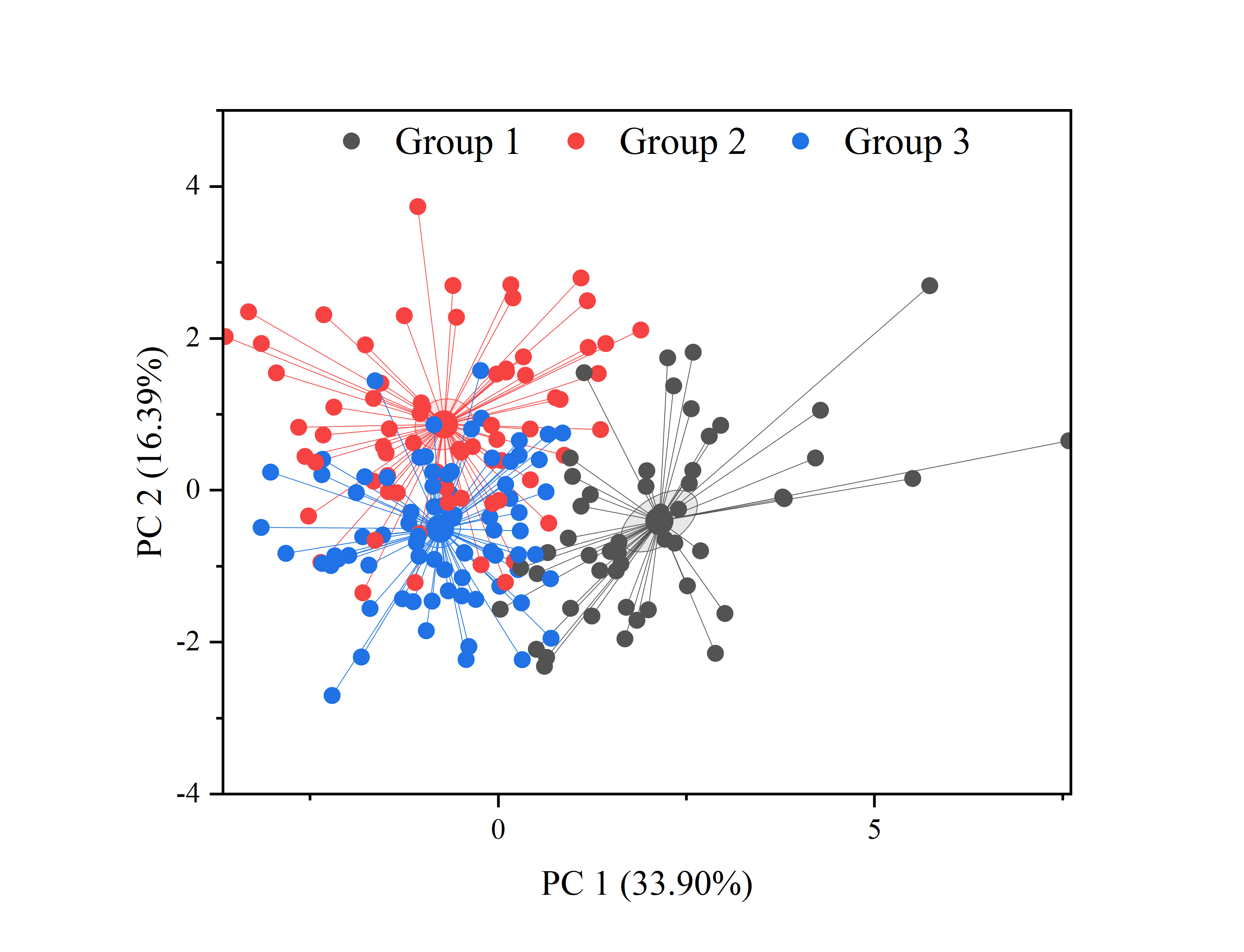


Figure S6 The cluster analysis of the 219 litchi resources


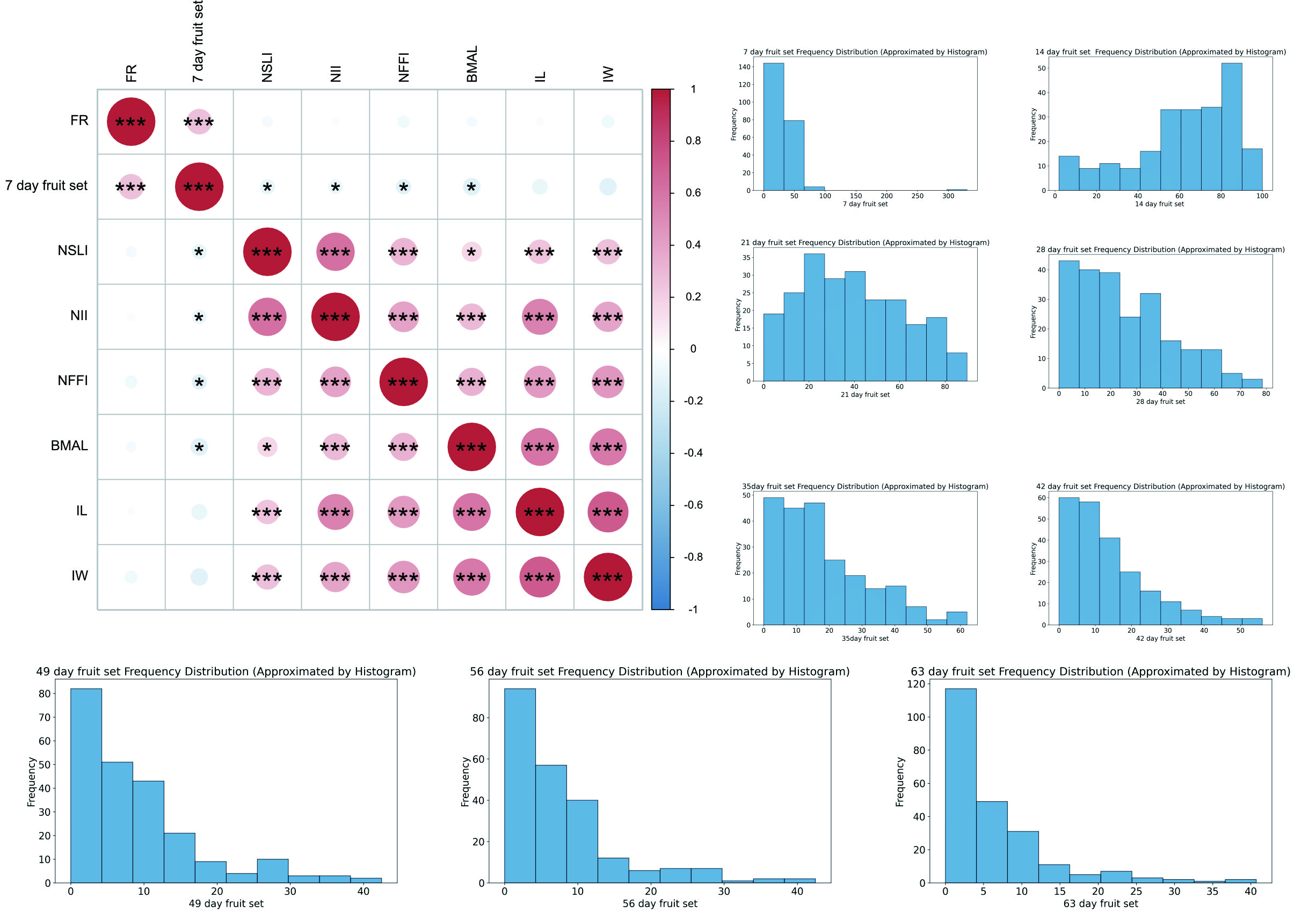


**Figure S7 Heatmap of correlation analysis between fruit set rate and various traits in 2022 and dynamic distribution of fruit set rate from day 7 to day 63**
